# Visuohaptic Feedback in Robotic-Assisted Spine Surgery for Pedicle Screw Placement

**DOI:** 10.3390/jcm14113804

**Published:** 2025-05-29

**Authors:** Giuseppe Loggia, Fedan Avrumova, Darren R. Lebl

**Affiliations:** Department of Spine Surgery, Hospital for Special Surgery, 523 East 72nd Street, New York, NY 10021, USA

**Keywords:** spinal fusion, robotic surgical procedures, minimally invasive surgical procedures, pedicle screws, torque, haptic feedback

## Abstract

**Introduction**: Robotic-assisted (RA) spine surgery enhances pedicle screw placement accuracy through real-time navigation and trajectory guidance. However, the absence of traditional direct haptic feedback by freehand instrumentation remains a concern for some, particularly in minimally invasive (MIS) procedures where direct visual confirmation is limited. During RA spine surgery, navigation systems display three-dimensional data, but factors such as registration errors, intraoperative motion, and anatomical variability may compromise accuracy. This technical note describes a visuohaptic intraoperative phenomenon observed during RA spine surgery, its underlying mechanical principles, and its utility. During pedicle screw insertion with a slow-speed automated drill in RA spine procedures, a subtle and rhythmic variation in resistance has been observed both visually on the navigation interface and haptically through the handheld drill. This intraoperative pattern is referred to in this report as a cyclical insertional torque (CIT) pattern and has been noted across multiple cases. The CIT pattern is hypothesized to result from localized stick–slip dynamics, where alternating phases of resistance and release at the bone–screw interface generate periodic torque fluctuations. The pattern is most pronounced at low insertion speeds and diminishes with increasing drill velocity. CIT is a newly described intraoperative observation that may provide visuohaptic feedback during pedicle screw insertion in RA spine surgery. Through slow-speed automated drilling, CIT offers a cue for bone engagement, which could support intraoperative awareness in scenarios where tactile feedback is reduced or visual confirmation is indirect. While CIT may enhance surgeon confidence during screw advancement, its clinical relevance, reproducibility, and impact on placement accuracy have yet to be validated.

## 1. Introduction

Spine surgery has progressed through successive technological innovations, with robotic-assisted (RA) systems contributing to greater precision, reproducibility, and procedural control [1,2]. Following the Food and Drug Administration’s (FDA) approval of the first robotic platform for navigated thoracolumbar pedicle screw placement in 2004, the role of robotics in spine surgery has expanded considerably [3]. Current systems function as shared-control platforms and offer features such as integrated navigation, trajectory guidance, and real-time feedback. Widely used systems include Mazor X Stealth (Medtronic), ExcelsiusGPS (Globus Medical), and ROSA (Zimmer Biomet), which differ in arm design, mounting method, and imaging compatibility. The selection is typically based on clinical goals, institutional infrastructure, and workflow integration [4,5,6].

### 1.1. Robotic Setup and Workflow

In RA spine surgery, procedural success relies on a systematic workflow built around imaging integration, secure patient setup, and precise navigation. The surgical plan begins with the acquisition of high-resolution preoperative computed tomography (CT) scans, which are imported into the robotic system to map out individualized screw trajectories. To maintain positional accuracy throughout the operation, the robotic platform is anchored firmly to either a stable anatomic landmark, such as the posterior superior iliac spine, or directly to the operating table or floor, depending on the system specifications [7]. During setup, preoperative imaging is aligned with the patient’s anatomy through a registration process using fluoroscopic verification and optical tracking. A dynamic reference frame is affixed to the patient to maintain the validity of registration throughout the procedure. Following registration, the robotic arm is deployed to guide the instruments along the preplanned paths [8].

**Figure 1:**
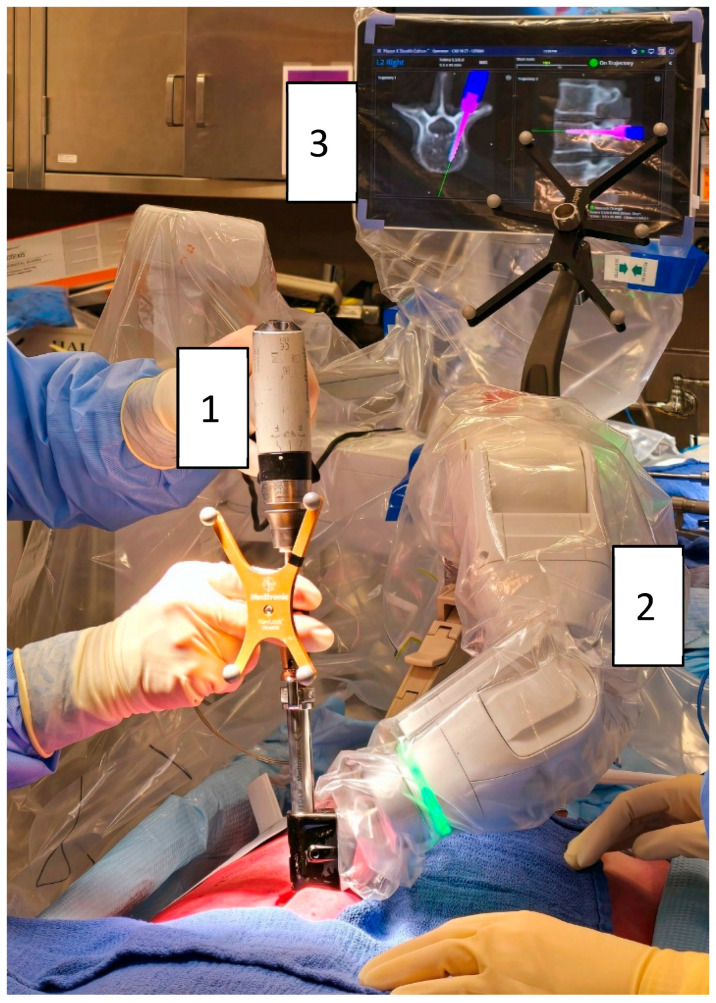
Robotic-assisted pedicle screw insertion using the Mazor X^®^ system (Medtronic): (1) Automatic screwdriver (Powerease™ driver with optical tracking). (2) Robotic arm mounted on the table and the posterior superior iliac spine. (3) Robotic interface displaying screw advancement (pink).

In the screw placement workflow, trajectory verification is first performed with a navigated probe, followed by the creation of a pilot channel using a high-speed drill. In select cases, a Kirschner wire may be placed to assist with trajectory stabilization and enhance haptic feedback, while tapping of the pilot tract is recommended to minimize the risk of skiving. Once the tract is prepared, screw insertion is performed either with powered assistance or manually. As the screw advances, the robotic interface continuously displays positional data (Figure 1) [3,5,6,9].

### 1.2. Clinical Utility

RA spine surgery has been associated with improved clinical outcomes and enhanced patient safety through greater precision and workflow standardization. By enabling more accurate pedicle screw placement, RA platforms can reduce the risk of malposition-related complications [3,10]. Additionally, decreased reliance on intraoperative fluoroscopy and the elimination of CT-based navigation contribute to lower radiation exposure for both patients and surgical teams [11,12]. Their ability to stabilize instrument trajectories, reduce operator tremors, and navigate complex anatomical structures has further expanded the role of RA systems in minimally invasive (MIS) spine surgery [7,13,14]. These features may reduce soft tissue disruption, intraoperative blood loss, and operative duration, with potential downstream benefits such as shorter hospital stays and improved surgical efficiency [7,14]. While initial capital and implementation costs remain high, these may be offset over time by lower revision rates, reduced perioperative morbidity, and decreased overall hospitalization costs [2,15].

### 1.3. Technical Considerations

Despite its advancements, RA spine surgery remains subject to technical limitations, including registration errors and trajectory deviations that may compromise accuracy. Skiving and intraoperative motion further contribute to these complexities [12,16,17]. These factors may lead to subtle post-registration frame shifts, wherein deviations in screw placement are not detected by the robotic platform despite the system’s precision. As a result, there remains a risk that procedural accuracy may be erroneously assumed based solely on the maintained appearance of navigation alignment, potentially masking underlying deviations [18]. RA systems have demonstrated high placement fidelity; however, there is a perception by some surgeons that RA surgery lacks haptic feedback, which may limit intraoperative awareness. This concern is particularly relevant in MIS cases, in which direct visualization is limited, potentially requiring the need for additional radiographic verification of screw positioning. Broader acceptance of robotic assistance in spine surgery may be supported by the availability of real-time visual or haptic cues that mirror the tactile experience of freehand techniques. A perceived disconnect between surgeon input and screw advancement can pose a barrier to adoption, especially among those who value hands-on feedback during instrumentation [1,19,20].

This challenge has been recognized in previous research, leading to efforts aimed at integrating haptic feedback into RA pedicle screw placement. Nakdhamabhorn et al. investigated a robotic system with sensorless haptic feedback to improve haptic perception in RA spine surgery [21]. However, the study’s preliminary nature, with only four screw placements, limits the generalizability of its findings and underscores the need for further validation before broader clinical implementation.

### 1.4. Purpose

This technical note describes a pragmatic approach to recognizing whether a screw is seated within bone in RA spine surgery. The method utilizes a slow-speed automated drill, leverages a visuohaptic resistance and a distinct “cyclical insertional torque” (CIT) pattern, and offers an intuitive and reproducible intraoperative indicator of proper screw engagement.

## 2. Technical Note

The described CIT pattern is derived from extensive clinical experience at a high-volume academic institution, where RA spine surgery has been a core component of surgical practice. The surgeon, with six years of experience in RA spine surgery, has performed more than 600 pedicle screw placements annually in recent years, predominantly using the Mazor X^®^ platform (Medtronic). In addition, the CIT pattern was consistently observed by spine research fellows during cadaveric procedures, suggesting that the phenomenon may be perceptible beyond individual user experience. However, no quantitative torque measurements were recorded in this report, and the current description is based on observational findings. As this technical note presents an early intraoperative observation, further objective validation is planned to assess reproducibility and clinical relevance.

### 2.1. “Cyclical Insertional Torque” Pattern

CIT is a phenomenon observed during slow-speed pedicle screw insertion using an automated drill in RA spine surgery. This pattern is characterized by a cyclical, rotational movement of the screw as it advances into bone rather than a continuous linear advance. This phenomenon becomes visually evident on the navigation screen, where the displayed screw trajectory mirrors the subtle circular motion of the screw within the pedicle. Additionally, the oscillations can be tactilely perceived when holding the drill, reinforcing the notion that the screw engages with alternating regions of resistance and compliance within the bone matrix.

**Figure 2:**
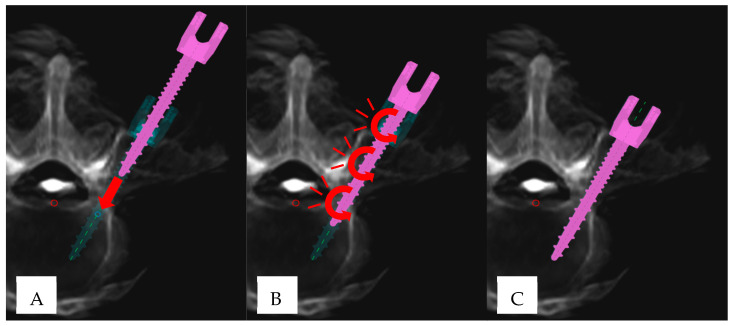
“Cyclical insertional torque” (CIT) pattern in robotic-assisted pedicle screw placement: (**A**) Initial phase of pedicle screw insertion (pink), demonstrating linear advancement (red arrow) until the screw tip is fully seated within the pedicle. The turquoise trajectory represents the preplanned screw position. (**B**) Manifestation of CIT, characterized by subtle oscillatory/circular movements (red curved arrows). (**C**) Final screw position, aligning with the preplanned trajectory.

This pattern is not present during high-speed insertion, suggesting that the speed of insertion plays a critical role in its manifestation. CIT was consistently observed at insertional speeds between 20 and 50 RPM, with lower speeds producing the most distinct expression. The pattern remained visible up to approximately 70 RPM but diminished progressively as speed increased. At higher insertion speeds, the interaction between the screw and the surrounding bone becomes more continuous, resulting in a predominantly linear advancement with diminished oscillatory behavior (Figure 2, Video S1).

### 2.2. Stick–Slip Effect

The stick–slip effect, a well-documented principle in tribology and mechanical systems, describes the alternating cycles of static friction (stick phase) and kinetic friction (slip phase) that occur during relative motion between two surfaces. This phenomenon emerges from the spontaneous depinning of mechanical contacts, driven by fluctuations in the local force landscape at the interface. As movement initiates, microscopic asperities at the contact surfaces alternately resist and release, causing the frictional force to oscillate between periods of accumulation and sudden discharge. These oscillations are governed by the complex interaction between elastic restoring forces within the materials and the heterogeneous distribution of local pinning forces, ultimately resulting in the characteristic intermittent motion observed in stick–slip systems [22,23,24,25].

In RA spine surgery, the CIT pattern observed during slow-speed pedicle screw placement is hypothesized to result from a localized stick–slip mechanism, wherein the screw appears to alternate between resistance and advancement as it interacts with the heterogeneous osseous microstructure. However, due to the continuous, power-induced advancement of the automatic drill and the pre-tapped trajectory, which follows a round path shaped by the screw’s cutting action, the progression is not characterized by abrupt sticking but rather by a smoother, cyclic movement. At low insertion velocities, the screw may experience intermittent phases of resistance and release, potentially contributing to cyclic torque variation. In contrast, at higher speeds, the screw is more likely to overcome micro-resistances within the bone structure too rapidly for significant oscillatory movement to develop, leading to a smoother and more continuous torque pattern [26,27].

In the stick phase, the applied pressure can compress trabecular structures, increasing local density and, consequently, resistance to further advancement. This temporary increase in frictional resistance leads to a buildup of rotational torque. Once the torque surpasses the static friction threshold, the stored energy is released, allowing the screw to advance [22,28]. In the slip phase, once the applied torque overcomes static resistance, the screw rapidly progresses into the next compliant region, where lower density facilitates smoother penetration. This transition from static to kinetic friction results in an oscillatory release of stored elastic energy, manifesting as cyclic variations in insertional torque [22,25].

### 2.3. Visuohaptic Feedback

The CIT pattern is perceptible through both visual and haptic sensory channels during RA pedicle screw placement. While the optical manifestation of CIT on the robotic display is readily apparent, the corresponding haptic feedback experienced by the surgeon is considerably subtler. The robotic navigation interface provides a real-time visual representation of the screw trajectory, displaying both its linear advancement and rotational behavior [10,29,30]. Haptic perception, in contrast, is conveyed through the surgeon’s hand as the drill transmits subtle variations in resistance. These fluctuations are perceptible but significantly less pronounced than the visual feedback on the robotic display.

### 2.4. Considerations for Manual Screw Insertion

In regions where suspected high-risk screw placement is anticipated due to anatomical, biomechanical, or system-related factors, manual screw insertion—akin to the freehand technique—is recommended to enhance trajectory control and mitigate deviations. These high-risk placement zones encompass conditions that may compromise screw accuracy, including registration errors, trajectory deviations, intraoperative motion, and skiving. However, while the CIT pattern is no longer recognized in manual insertion, haptic feedback is significantly heightened. This is due to the direct mechanical coupling between the surgeon’s hand and the screw through the manual screwdriver, unfiltered by the rotational inertia and torque regulation of an automated drill.

## 3. Discussion

The CIT pattern represents the first described intraoperative indicator in RA spine surgery that may enhance both visual and haptic feedback without necessitating additional instrumentation. Its recognition during slow-speed pedicle screw insertion could serve as a supplementary cue for bone engagement, potentially enhancing intraoperative awareness alongside the visual guidance provided by RA platforms. At present, there is no evidence to confirm that CIT improves accuracy, efficiency, or safety, and its recognition is based on subjective perception.

### 3.1. Potential Factors Influencing CIT Expression

While the precise determinants of CIT remain to be fully established, several factors may contribute to its manifestation. This includes thread design, pitch, outer and root diameter, shapes, and screw engagement with osseous structures, and may influence the oscillatory pattern observed during insertion. The outer diameter and pitch may modulate torque fluctuations during insertion, influencing the perceptibility of CIT [31,32]. These structural elements may modulate torque fluctuations during insertion, influencing the perceptibility of CIT. Insertion speed also appears to influence CIT expression, with preliminary observations suggesting that higher drill speeds may reduce or eliminate the pattern’s visibility. This aligns with the mechanical principles of the stick–slip phenomenon, as described by Oprișan et al., where increasing sliding velocity transitions an initially discontinuous stick–slip effect into a linear motion [33]. Applying these findings to pedicle screw insertion, it is conceivable that at lower speeds, the interaction between the screw and bone surface induces periodic resistance variations, manifesting as CIT [22].

Bone density may also play a role in CIT expression, as the mechanical properties of the surrounding osseous structures influence the resistance encountered during screw insertion. Denser trabecular bone, characterized by higher mineralization and structural integrity, likely increases the magnitude of torque fluctuations, potentially amplifying CIT. In contrast, osteoporotic or low-density bone may offer less resistance, diminishing the perceptibility of CIT due to a more uniform insertion trajectory [34,35]. This relationship aligns with prior studies on screw fixation, where increased bone density has been associated with higher insertional torque and improved mechanical stability [36]. Additionally, surface coatings that modify the frictional interface and the precise positioning of the screw within the pedicle—whether engaging more cortical or cancellous bone—may further impact torque variability and CIT expression [34,37,38]. However, the extent to which bone density directly modulates CIT remains uncertain, warranting further investigation.

### 3.2. Stick–Slip Dynamics in CIT

The underlying mechanism of CIT may be explained by the principles of stick–slip dynamics, a well-documented phenomenon in frictional systems. An analogy for understanding this process can be drawn from the resistance encountered when dragging sandpaper across a carpet, where intermittent transitions between static and kinetic friction create an oscillatory motion [23]. Similarly, during pedicle screw insertion, alternating phases of engagement and release at the bone–screw interface may give rise to CIT, governed by localized stick–slip effects.

Recent advances in physics-informed neural networks and deep learning (DL) models have shown promise in extracting frictional parameters from stick–slip datasets. Borate et al. demonstrated its ability to recognize characteristic frictional transitions, suggesting its potential application in CIT analysis [25]. By training DL models on CIT data, it may be possible to develop intraoperative algorithms capable of identifying torque signatures indicative of successful in-bone placement versus misaligned trajectories.

### 3.3. Visuohaptic Feedback

Recognition of CIT during RA screw placement appears to rely on a combination of visual and haptic sensory input, both of which may contribute to intraoperative perception of the pattern. Unlike traditional freehand techniques, where tactile sensation plays a dominant role, robotic systems modify the way sensory information is perceived by the surgeon. In this context, visual cues generated by the robotic navigation interface often become more prominent, while haptic inputs may be attenuated by the mechanical properties of the powered instrumentation. Understanding the factors that influence the relative dominance of visual versus haptic feedback is essential in appreciating how CIT is perceived intraoperatively.

This difference may be attributed to several mechanisms. Xu et al. demonstrated that visual information is typically processed before haptic feedback, aligning with weight perception theories wherein early visual cues guide subsequent motor responses [39]. Additionally, as the screw advances, the robotic digital tracking systems generate a seamless, uninterrupted depiction of the screw’s movement, effectively enhancing the visibility of oscillatory patterns [10,29,30]. Moreover, mechanoreceptors in the hand, such as Pacinian corpuscles and Merkel cells, mediate haptic feedback but may be influenced by mechanical dampening from the drill system and sensory adaptation [40,41,42]. As neural responsiveness decreases with sustained stimuli, repetitive torque fluctuations become less perceptible, further emphasizing the dominance of visual feedback in CIT recognition [43].

### 3.4. Limitations

While the CIT pattern presents a promising intraoperative indicator in RA spine surgery, several factors constrain its consistency and clinical applicability. The robotic system used does not provide real-time torque output, and although a compatible torque meter exists, it is not FDA-approved or sterilizable, precluding its use in the operating room. The CIT observations described are based on consistent intraoperative recognition by the lead surgeon and independent observers, but they remain subjective in the absence of recorded torque data. Moreover, its expression may be influenced by surgical conditions, anatomical variability, and the surgeon’s perceptual acuity, raising questions about its reliability across different scenarios. These concerns may be especially relevant in high-stress operative environments or when used by less experienced surgeons, who may need to focus their sensory attention on other intraoperative factors such as skive prevention or soft tissue pressure mitigation. Notably, the behavior of CIT in cases of minimal cortical breaches and subtle trajectory deviations remains undefined, underscoring the need for further investigation into its biomechanical underpinnings and clinical significance. This study reflects a single-surgeon technical observation at one institution. Although CIT has also been consistently noted in cadaveric procedures by other team members, it has not yet been evaluated in a formal, multi-surgeon or multi-center setting. As such, its generalizability, interobserver reliability, and reproducibility remain uncertain. Additionally, CIT recognition is inherently subjective, relying on the surgeon’s real-time perception without standardized thresholds or objective parameters. Variability in haptic sensitivity and visual processing may introduce inconsistencies, particularly in high-paced surgical environments where visual feedback is the primary guidance modality. To enhance the objectivity and clinical utility of the CIT pattern, future research should prioritize the development of quantitative intraoperative assessment tools, such as real-time torque analysis and automated pattern recognition algorithms, capable of detecting characteristic torque fluctuations indicative of optimal screw placement.

## 4. Outlook

The future trajectory of RA spine surgery is expected to encompass both the expansion of procedural indications and ongoing refinements addressing current technological limitations. Early clinical experience has demonstrated measurable improvements in workflow efficiency and screw placement accuracy during the initial learning curve, reflected by reductions in registration times and consistently high rates of accurate screw placement. Progressive enhancements in technique, instrumentation, and operating room familiarity have further contributed to these outcomes, although the number of cases required to achieve proficiency remains a subject of ongoing investigation [7,44,45,46,47]. While early adoption has primarily focused on pedicle screw placement, advancements in robotic technology suggest a growing role in decompressive procedures, percutaneous interventions, and complex spinal reconstructions, where precise navigation and soft tissue preservation are paramount [10,48]. Future iterations of robotic platforms are anticipated to incorporate enhanced registration algorithms capable of adapting to soft tissue structures and multimodal imaging inputs, including magnetic resonance imaging [29,49]. As these innovations progress, continued clinical research will be essential to delineate the benefits, limitations, and optimal utilization of RA systems in modern spine surgery.

### Future Directions of “Cyclical Insertional Torque”

As RA spine surgery continues to evolve, efforts should focus on enhancing intraoperative awareness and feedback to match the intuitive control traditionally associated with freehand techniques. Unlike freehand techniques, where tactile input forms an integral part of screw insertion, RA workflows may alter the sensory experience during instrumentation [1,19,20]. Within this context, the observation of CIT offers a potential adjunct to support intraoperative awareness by providing a visuohaptic cue during screw advancement. Future investigations are needed to systematically characterize CIT patterns and establish their relationship with optimal bone engagement. As an initial step, a cadaveric study utilizing torque-sensing instrumentation may help characterize baseline CIT waveforms under conditions approximating optimal screw placement. This could be followed by the purposeful introduction of suboptimal trajectories such as cortical breaches, angular deviations, or insertion into osteopenic bone to explore potential variations in CIT expression. These preliminary findings may then inform prospective clinical studies involving multiple surgeons and institutions to assess reproducibility, interobserver consistency, and potential clinical relevance. Integrating torque-sensing technologies with machine learning-based recognition models may further enhance intraoperative decision-making without adding procedural complexity [25]. If confirmed, CIT may represent an additional tool for augmenting surgeon confidence and maintaining precision in RA spine surgery.

## 5. Conclusions

CIT is a newly described intraoperative observation that may provide visuohaptic feedback during pedicle screw insertion in RA spine surgery. Through slow-speed automated drilling, CIT offers a cue for bone engagement, which could support intraoperative awareness in scenarios where tactile feedback is reduced or visual confirmation is indirect. However, its clinical utility remains to be fully established, as factors such as anatomical variability, insertion speed, and bone density may influence its expression.

## Data Availability

No data were generated or analyzed in this study.

## Supplementary Materials

The following supporting information can be downloaded at https://www.mdpi.com/article/10.3390/jcm14113804/s1: Video S1: Intraoperative visualization of the “cyclical insertional torque” (CIT) pattern during robotic-assisted placement of an L5 pedicle screw. The video demonstrates insertion using a slow-speed handheld drill, with the robotic interface displaying rhythmic oscillatory screw motion consistent with CIT.

## Abbreviations

The following abbreviations are used in this manuscript:

RA	Robotic-assisted
MIS	Minimally invasive
CIT	“Cyclical insertional torque”
FDA	Food and Drug Administration
CT	Computed tomography
DL	Deep learning
